# A High-Density Genetic Linkage Map and Fine Mapping of QTL For Feed Conversion Efficiency in Common Carp (*Cyprinus carpio*)

**DOI:** 10.3389/fgene.2021.778487

**Published:** 2021-11-12

**Authors:** Xiaofeng Zhang, Peixian Luan, Dingchen Cao, Guo Hu

**Affiliations:** National and Local United Engineering Laboratory for Freshwater Fish Breeding, Heilongjiang River Fisheries Research Institute, Chinese Academy of Fishery Sciences, Harbin, China

**Keywords:** common carp (*Cyprinus carpio*), genetic linkage map, feed conversion efficiency, QTL mapping, candidate genes

## Abstract

Feed conversion efficiency (FCE) is an economically crucial trait in fish, however, little progress has been made in genetics and genomics for this trait because phenotypes of the trait are difficult to measure. In this study, we constructed a high-density and high-resolution genetic linkage map with 28,416 SNP markers for common carp (*Cyprinus carpio*) based on high throughput genotyping with the carp 250K single nucleotide polymorphism (SNP) array in a full-sib F_1_ family of mirror carp (*Cyprinus carpio*) consisting of 141 progenies. The linkage map contained 11,983 distinct loci and spanned 3,590.09 cM with an average locus interval of 0.33 cM. A total of 17 QTL for the FCE trait were detected on four LGs (LG9, LG20, LG28, and LG32), explaining 8.9–15.9% of the phenotypic variations. One major cluster containing eight QTL (qFCE1-28, qFCE2-28, qFCE3-28, qFCE4-28, qFCE5-28, qFCE6-28, qFCE7-28, and qFCE8-28) was detected on LG28. Two clusters consisting of four QTL (qFCE1-32, qFCE2-32, qFCE3-32, and qFCE4-32) and three QTL (qFCE1-20, qFCE2-20, and qFCE3-20) were detected on LG32 and LG20, respectively. Nine candidate genes (*ACACA*, *SCAF4*, *SLC2A5*, *TNMD*, *PCDH1*, *FOXO*, AGO1, *FFAR3,* and *ARID1A*) underlying the feed efficiency trait were also identified, the biological functions of which may be involved in lipid metabolism, carbohydrate metabolism, energy deposition, fat accumulation, digestion, growth regulation, and cell proliferation and differentiation according to GO (Gene Ontology). As an important tool, high-density and high-resolution genetic linkage maps play a crucial role in the QTL fine mapping of economically important traits. Our novel findings provided new insights that elucidate the genetic basis and molecular mechanism of feed efficiency and the subsequent marker-assisted selection breeding in common carp.

## Introduction

Aquatic products are an important source of nutrition for people around the world, especially in food-deficient countries (Easterling, 2007; Rice and Garcia, 2011). The long-term challenge for aquaculture breeders is to improve the yield-related traits in fish to meet the growing demand for fish products while minimizing their impact on the environment (Wringe et al., 2010; Laghari et al., 2013). As feed cost comprises about 65–75% of the total production cost in most aquaculture industries, an effective way to solve this problem is to breed fish with high feed conversion efficiency (FCE) (Gjedrem and Baranski, 2009). Generally, feed conversion efficiency is defined as the ratio of feed intake to weight gain in animals (Sherman et al., 2009; Ferreira et al., 2016). Similar to most yield-related traits, FCE is a heritable trait controlled by a series of genes, the environment, and their interactions, which has been confirmed in livestock, poultry, and fish (Herd et al., 2003; Cai et al., 2008; Begli et al., 2016; Lu et al., 2017; Fu et al., 2020; Miao et al., 2021). So, the aim of improvement in FCE could be achieved by breeding to select genetically superior animals.

Globally, as one of the most important fishes, common carp (*Cyprinus carpio*) is cultured in over 100 countries worldwide with over 4 million metric tons of global annual production (Bostock et al., 2010; http://www.fao.org/fishery/statistics/global-aquaculture-production/query/en). Because of the economic importance of common carp for the aquaculture industry, over the past decades, researchers have developed a variety of genomic resources and genetic tools to facilitate genetic improvement and breeding programs (Sun and Liang, 2004; Christoffels et al., 2006; Williams et al., 2008; Xu P. et al., 2014). Especially, the completion of genome sequencing of Songpu mirror carp and subsequent development of a 250,000 high-quality SNPs array chip which provides favorable tools for ultra-high density linkage map construction (Xu J. et al., 2014; Xu P. et al., 2014; Xu et al., 2019).

High-quality genetic linkage maps are essential tools for quantitative trait loci (QTL) mapping. In past decades, a number of linkage genetic map had been constructed and QTL mapping for many traits in the fish have been studied (Yue, 2014; Ashton et al., 2017). Common carp genetic researchers have also constructed a number of linkage maps in common carp based on different mapping families using SSR and SNP markers in recent two decades. These linkage maps have been widely used for QTL mapping of many economically important traits in common carp. Such as growth-related traits, disease resistance, meat quality, sex-determination traits, and so on have been successfully mapped (Jun Wang, 2012; Laghari et al., 2013; Kuang et al., 2015; Lv et al., 2016; Peng et al., 2016; Zheng et al., 2016; Jia et al., 2020; Su et al., 2020).

It is obvious that genetic improvements for the efficiency of feed utilization are important. So far, some DNA variants that play a role in the feed efficiency of poultry and livestock have been proposed by QTL mapping and association studies (Koning et al., 2003; Barendse et al., 2007; Sherman et al., 2009; Do et al., 2014; Mignon-Grasteau et al., 2015; Silva et al., 2019; Ye et al., 2020; Delpuech et al., 2021). However, the feed intake of each experimental individual is generally difficult to measure in fish. So, only a few QTL analyses on FCE traits have been conducted in fish and these QTL mapping studies may be insufficient because of the relatively low power of linkage analyses (Wang et al., 2012b; Lu et al., 2017; Barría et al., 2021). Recently, with the aid of the high throughput SNP genotyping array, an ultra-high density linkage map has revealed many important findings related to growth-related traits, sex dimorphism, and muscle quality traits in common carp (Peng et al., 2016; Zheng et al., 2016).

In the present study, using a 250,000 SNPs chip, a high-density linkage map with 28,416 SNP markers was constructed in a full-sib family of common carp, which is the highest density genetic linkage map for common carp so far. Using this map, we carried out the QTL fine mapping for the FCE trait. The candidate genes were also recognized from the genome regions of quatative trait loci. Furthermore, the results of our analysis wil provide a basis for genetically improving the feed efficiency of common carp in the future.

## Materials and Methods

### Mapping Family and Phenotypic Measurements

Songpu mirror carp (SMC) is a strain derived from a European subspecies (*C. carpio*) of common carp, which is one of the most valuable fish species for freshwater breeding as well as one of the species that is highly promoted to culture in China. An F_1_ full-sib family of SMC was produced at the Hulan Aquaculture Experimental Station of the Heilongjiang River Fisheries Research Institute, Harbin, China. A large number of Songpu mirror carp individuals (*n* = 500) were collected as brood fish, and their genetic distances were estimated using polymorphic microsatellite markers. Then a pair of female and male mature fish with suitable genetic distance were used as the maternal parent and the paternal parent to generate the experiment family (F_1_) by artificial crossing. After hatching, approximately 3,000 offspring were raised in a pond under a standard feeding regime. A total of 150 progeny were randomly collected from the experimental population as the fish panel for feed conversion tests after 60 days post-hatching. The fish were stocked individually in a tank with a size of 90 cm × 85 cm × 70 cm in a series of re-circulating aquarium systems. All conditions in these tanks were regularly maintained throughout the experiment, i.e., water temperature was 22°C and water flow rate is 1 ms^−1^. The juveniles were fed solely by complete carp extruded feed. Each experimental fish was equipped with a feed box. The pallet feeds used in this experiment contain 34% crude protein, 10% crude lipid, and 7% ash (Supplementary Table S1). In order to eliminate potential differences and as much as possible, we trained the experimental fish to adapt to the culture environment and reared them individually in the re-circulating aquarium tanks for 2 weeks before the feed conversion trial. During the feed conversion trail, the experimental family was fed three times (9:00, 13:00, and 17:00) a day. The feces in each tank were siphoned out daily and the water was changed completely once a week. The residue of feed was siphoned out, recorded, and deducted from the feed weight supplied each day to acquire the accurate feed consumption of each fish.

Subsequent phenotypic measurements of feed conversion efficiency were made. Phenotypic data of the FCE were collected after 3 months of feeding trials. Briefly, we recorded body weight (BW) at the beginning (initial BW, BWI) and the end (final BW, BWF) of the feeding test. Total feed intake (FI) was recorded as the difference between the beginning and final weight of feed used during the test. Then, the FCE was calculated as the BW gain after the experiment divided by total FI. Since the phenotypically extreme or abnormal individuals were excluded from the experiment trial according to the statistical analysis, a total of 141 offspring was determined as the experimental sample for linkage map construction and QTL analysis.

### DNA Extraction and SNP Genotyping

Approximate 0.5 ml of blood from each sample was collected into a tube containing EDTA. Genomic DNA was extracted from the preserved blood using QIAamp DNA BloodMini Kit (Qiagen, Shanghai, China) following the manufacturer’s protocol. A NanoDrop-1000 spectrophotometer (Thermo Fisher Scientific, United States) was used to determine the DNA concentration in each sample and the integrity of DNA was checked by 1.5% agarose gel electrophoresis. DNA samples used for genotyping were diluted to 50 ng/ul and genotyped at GeneSeek (Lincoln, Nebraska, United States) using the common carp 250K SNP array (Xu J. et al., 2014). Affymetrix CEL files were analyzed using Affymetrix Genotyping Console software (version 4.0) for quality control and genotype calling. The CHP files generated from Affymetrix Genotyping Console were then extracted and converted to Ped/Map format for further analysis. SNPs were removed if they had a missing genotype rate >1% and a minor allele frequency (MAF) < 1%. SNPs retained were collected for subsequent analysis.

### Linkage Map Construction

Prior to map construction, Mendelian inheritance errors also were checked by a chi-square test using the parameters of segregation distortion (*p* < 0.001). Only the SNPs conforming to Mendelian inheritance were used for further linkage analysis. Then, the remaining markers through a series of quality control procedures above were subjected to JoinMap for linkage map construction. The double pseudo-test cross strategy was employed for linkage analysis. SNPs were separated into three segregation patterns: AAxAB or BBxAB (1:1 segregation only in male parent), ABxAA or ABxBB (1:1 segregation only in female parent), and ABxAB (1:2:1 segregation in both parents). The linkage maps were constructed by using JoinMap 4.0 (Van Ooijen, 2006) with “CP” type population, which is designed to handle F_1_ population data containing various genotype configurations. A threshold of 5.0 was set for assigning markers into different linkage groups (LGs). The Kosambi mapping function was used to estimate map distances in centiMorgans (cM) through the maximum likelihood (ML) algorithm. Graphical visualization of the linkage maps was drawn by MapChart 2.2 software (Voorrips, 2002).

### QTL Mapping and Annotation of Candidate Genes

QTL mapping analysis was performed for the FCE trait using software package MapQTL5.0 (Van Ooijen, 2004) with CIM (composite interval mapping) and MQM (multiple QTL model) mapping algorithms. A 1,000 permutation test was used to determine the LOD score significance thresholds at a 95% confidence level. After the 1,000 permutation test, a LOD threshold of 2.8–3.1 was set to identify significant QTL on each linkage group. The phenotypic variance explained (PVE) was estimated through stepwise regression (Li et al., 2007).

For each nearest SNP marker of QTL, we extracted candidate genes at the SNP loci from the reference genome of common carp. To annotate the functions of the FCE genes, we searched their orthologs by blastx against eudicots non-redundant database with an e-value threshold of 10^−5^. We also used Blast2GO (Conesa et al., 2005) with default parameters to assign the Gene Ontology (GO) to obtain more information of candidate genes that may be related to feed efficiency based on the annotation information.

## Results

### Phenotypic Data

Out of 150 experimental fish fed in individual tanks, 141 were alive throughout the 3 months of the experiment and used for the further phenotyped analysis of feed efficiency. The descriptive statistics of the phenotypic measurements of feed efficiency used for the present studies are given in Table 1. The panel had 26.52 ± 4.66 g mean initial body weight (BWI). The minimum and maximum for the BWI were 15.65 and 42.85 g, respectively. After the 3-month feeding trial, the average final body weight (BWF) of the individuals reached 150.72 ± 44.06 g. The minimum and maximum for the BWF were 42.99 and 271.52 g, respectively. The total feed intake (FI) of the fish was between 67.1 and 324.3 g with an average value of 200.1 ± 41.3 g. The deduced feed conversion efficiency (FCE) of the 141 individuals ranged from 40.8 to 89.4% with an average value of 60.8% (SD = 10.6%). The coefficient of variation (CV) of four traits ranged from 0.17 to 0.29 (Table 1).

**TABLE 1:** Descriptive statistics of phenotypic data.

Trait	Minimum	Maximum	Mean ± SD	CV
BWI (g)	15.65	42.85	26.52 ± 4.66	0.18
BWF (g)	42.99	271.52	150.72 ± 44.06	0.29
FCE (%)	40.8	89.4	60.8 ± 10.6	0.17
FI (g)	67.1	324.3	200.1 ± 41.3	0.21

FCE, feed conversion efficiency; BWI/BWF, initial/final body weight; FI, feed intake.

### Selection of SNP Markers

The genotypic data of all 141 F_1_ offspring and their parents is available. A total of 219,902 SNP markers were successfully genotyped. According to the assessment of genotyping quality and polymorphism in all samples from the mapping family, the genotypes of a total of 102,741 SNPs were exhibited polymorphic among the mapping panel. After further filtration with more stringent conditions to remove SNPs with low calling rate [SNPs calling rate 99% and minor allele frequency (MAF) greater than 1%], a total of 35,505 SNP markers were retained for further analysis. We selected 28,831 SNP markers based on segregation distortion and non-Mendelian inheritance (*p* < 0.001) for further linkage analysis and mapping (Table 2).

**TABLE 2:** SNPs selected for linkage mapping.

Item	Number
SNPs on array	24,9913
SNPs successful genotyped	219,902
Polymorphic SNPs	102,741
SNPs with high genotyping quality	35,505
SNPs used for linkage mapping after further filtering	28,831
SNPs mapped to linkage map	28,416

### Linkage Map Construction

**FIGURE 1:**
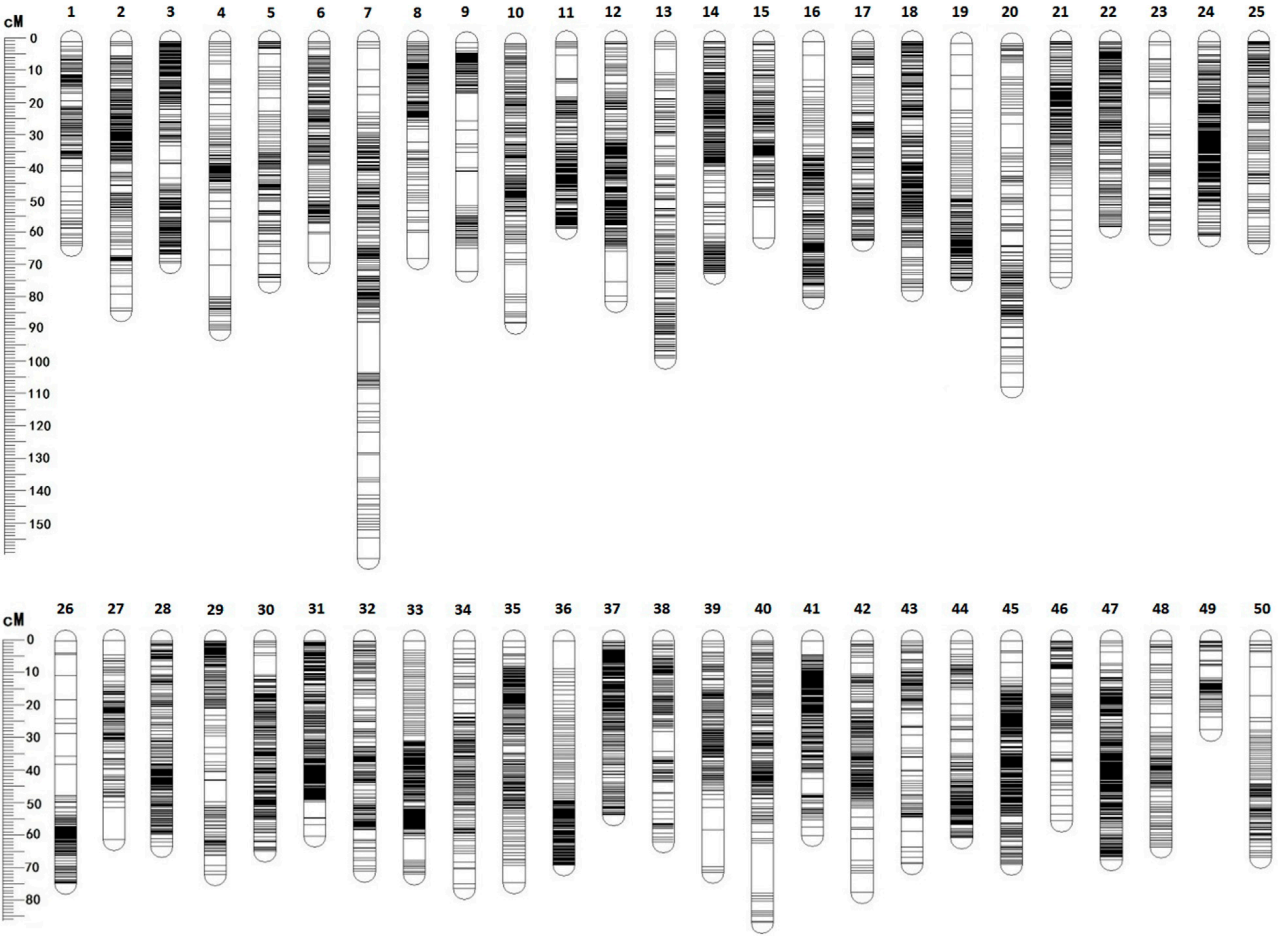
The high-density linkage genetic map for mirror carp.

**FIGURE 2:**
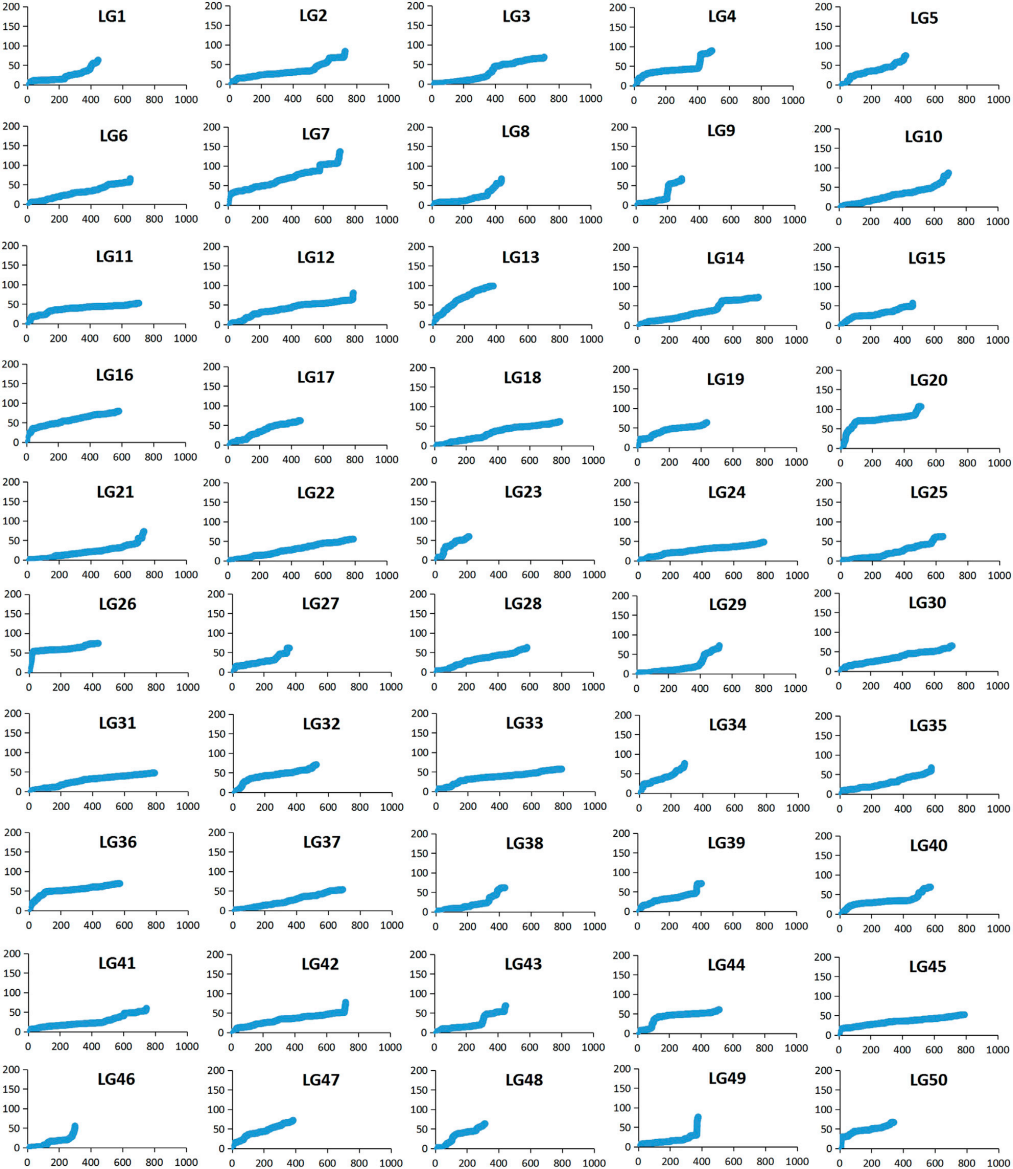
The patterns of marker distribution on each linkage group. The X-axis represents marker orders on each linkage group. The Y-axis represents SNP marker position (cM) or each linkage group.

Among 28,831 SNP markers, 28,416 SNPs were mapped on 11,983 distinct positions in 50 linkage groups (Figure 1; Table 3; Supplementary Table S2). A total of 98.6% of high-quality SNPs markers could be successfully mapped. The remaining 415 markers were not mapped. The total map distance was 3,590.09 cM with an average value of 71.80 cM. The number of markers on the linkage group varied from 213 (LG23) to 920 (LG45), and the average number of mapped markers per LG was 568 markers. The genetic length of each LG ranged from 53.48 cM (LG37) to 159.93 cM (LG7) with an average length of 71.80 cM. The average locus intervals varied from 0.14 cM in LG31 to 0.58 cM in LG7. The overall average marker interval was 0.33 cM. Based on the method described by Chakravarti et al. (1991), the expected genome length was estimated to be 3,623.71 cM. So, the percentage of the genome covered by the linkage map was calculated to be 99.07%. Detailed information and characteristics of this high-density genetic map were summarized in Table 3. As shown in Figure 2, the SNP distribution on each linkage group was also examined, which illustrated an even distribution of SNP markers on each linkage group with some exceptions at the middle and terminal regions of LGs.

**TABLE 3:** Summary of the linkage map of the mirror carp.

LG	No. of SNPs	Distinct positions	Genetic length (cM)	Locus interval (cM)
1	444	153	63.14	0.41
2	726	279	83.48	0.30
3	703	296	68.48	0.23
4	489	157	89.21	0.57
5	417	154	74.39	0.48
6	647	206	68.51	0.33
7	730	276	159.93	0.58
8	439	164	67.22	0.41
9	291	137	70.76	0.52
10	691	274	86.61	0.32
11	817	308	57.75	0.19
12	791	337	80.55	0.24
13	382	207	98.09	0.47
14	758	305	71.76	0.24
15	467	186	60.74	0.33
16	582	254	79.46	0.31
17	457	200	61.54	0.31
18	873	414	77.04	0.19
19	437	199	73.42	0.37
20	507	198	106.16	0.54
21	735	250	73.06	0.29
22	865	308	57.33	0.19
23	213	108	59.75	0.55
24	847	388	60.14	0.16
25	645	235	62.38	0.27
26	430	189	74.39	0.39
27	361	150	61.11	0.41
28	583	260	63.05	0.24
29	521	198	71.75	0.36
30	714	286	64.56	0.23
31	858	438	59.99	0.14
32	525	242	70.72	0.29
33	862	386	71.56	0.19
34	288	160	76.03	0.48
35	576	302	74.21	0.25
36	572	258	68.84	0.27
37	691	333	53.48	0.16
38	436	196	61.66	0.31
39	403	193	71.10	0.37
40	572	282	68.58	0.24
41	745	323	59.80	0.19
42	722	254	77.35	0.30
43	450	177	68.35	0.39
44	515	209	60.55	0.29
45	920	391	68.53	0.18
46	302	138	55.27	0.40
47	384	227	72.26	0.32
48	314	126	63.22	0.50
49	378	136	76.37	0.56
50	341	136	66.49	0.49
Total	28,416	11,983	3,590.09	0.33

### QTL Mapping

**FIGURE 3:**
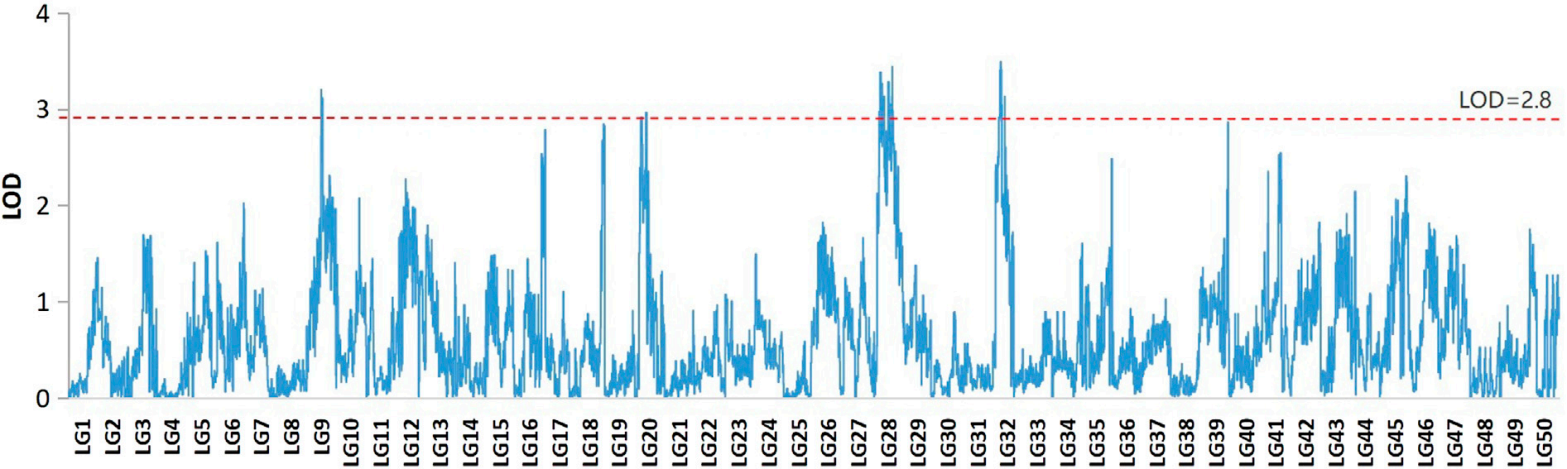
QTL mapping and significant regions were identified for FCE in common carp.

**FIGURE 4:**
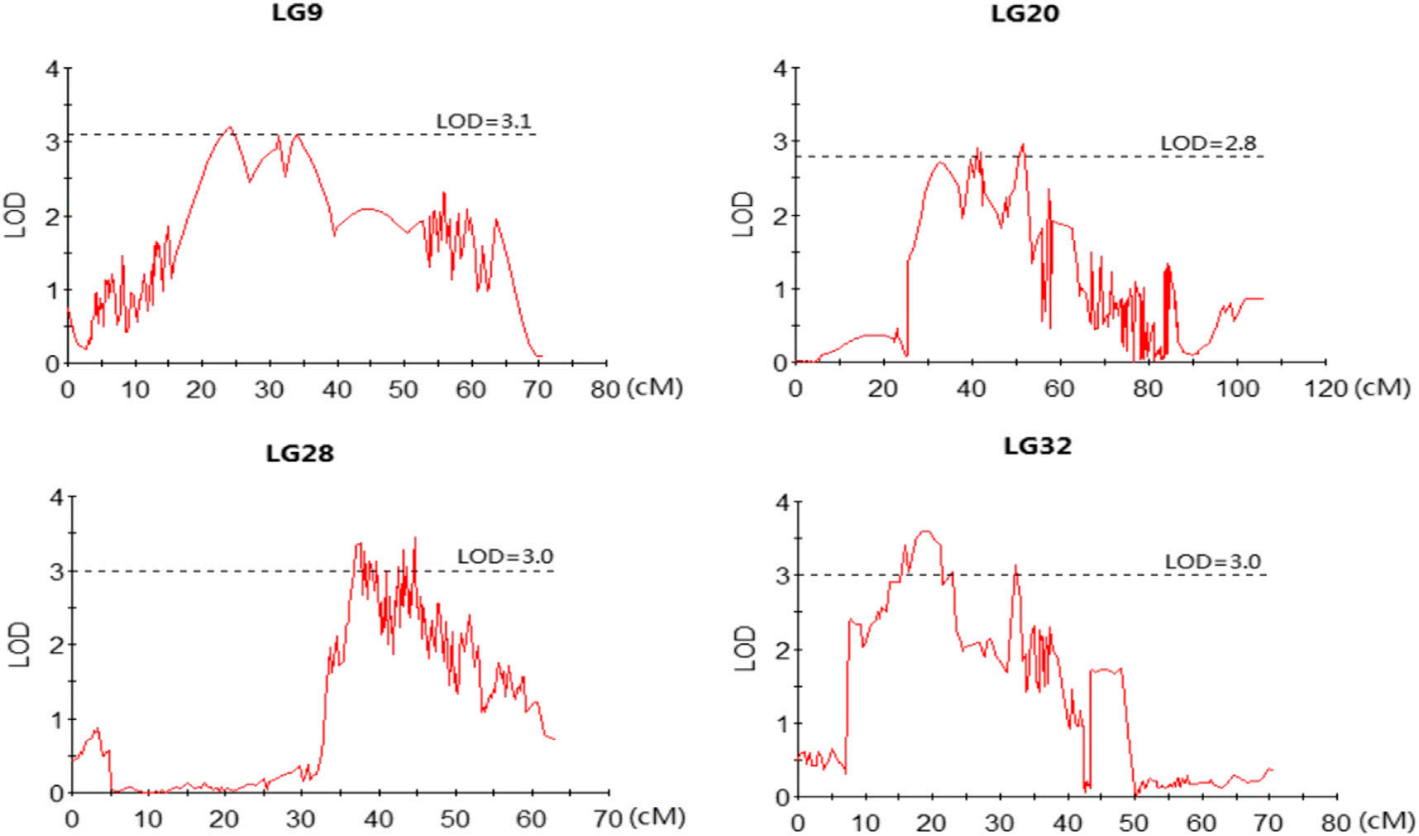
LOD curves for QTLs contributing to FCE. The X-axis indicates marker distance and the Y-axis represents the LOD with the dashed line indicating the threshold value of permutation.

The profiles and characteristics of the QTL associated with FCE are presented in Table 4; Figures 3, 4. A total of 17 significant QTL regions were mapped onto four LGs using composite interval mapping (CIM) and multiple QTL models (MQM) in MapQTL 5.0 program. The LOD significance thresholds for the FCE ranged from 2.8 to 3.1 based on the permutation test results. The phenotypic variances explained by each QTL (*R*_2_) were estimated by CIM. As shown in Table 3, these regions include 51 SNP loci, distributed on four LGs including LG9, LG20, LG28, and LG32 and having an effect of 8.9–15.9% phenotypic variance explained (PVE). Most of these QTL were clustered together on their respective LGs. One major cluster containing eight QTL (qFCE1-28, qFCE2-28, qFCE3-28, qFCE4-28, qFCE5-28, qFCE6-28, qFCE7-28, and qFCE8-28) was detected between the narrow position of 37.07–44.78 cM on LG28, accounting for 9.5–10.6% PVE. Among them, qFCE8-28 located at 44.52–44.78 cM had the highest LOD value (3.44) and correspondingly had the highest contribution to phenotypic variation (10.6%). On LG32, a cluster situated within a region (15.07–32.64 cM) consisted of four QTL (qFCE1-32, qFCE2-32, qFCE3-32, and qFCE4-32) with a LOD value of 3.04–3.49 and was able to explain 9.5–12.1% of the PVE. The most significant QTL qFCE2-32 located on LG32 at 16.29–20.45 cM presented the highest LOD value of 3.49, explaining 12.1% of the total PVE. Three QTL on LG20 (qFCE1-20, qFCE2-20, and qFCE3-20) consisted of a cluster located on 39.41–51.37 cM regions with LOD values of 2.85–2.96, and contributions to PVE of 8.9–9.2%. Two QTL (qFCE1-9 and qFCE2-9) on LG9 were detected at positions 15.81–27.08 and 32.32–38.81 cM, with LOD values of 3.20 and 3.11. The QTL qFCE2-9 located at LG9 at 32.32–38.81 cM explained the highest percentage of the total PVE of 15.9%.

**TABLE 4:** Analysis of QTL and estimation of genetic effects.

Linkage group	QTL name	Marker interval	Marker interval (CM)	Linkage group size (CM)	LOD	Permutation*	PVE%	Nearest maker
LG9	qFCE1-9	snp108444-snp022617	15.81–27.08	70.76	3.20	3.1	12.9	snp204986
LG9	qFCE2-9	snp105119-snp098735	32.32–38.81	70.76	3.11	3.1	15.9	snp057963
LG20	qFCE1-20	snp049074-snp067360	39.41–41.65	106.16	2.91	2.8	9.1	snp221731
LG20	qFCE2-20	snp041990-snp145914	41.81–42.35	106.16	2.85	2.8	8.9	snp145912
LG20	qFCE3-20	snp145904-snp146285	49.45–51.37	106.16	2.96	2.8	9.2	snp145909
LG28	qFCE1-28	snp247225-snp164238	37.07–37.90	63.05	3.38	3.0	10.4	snp247112
LG28	qFCE2-28	snp060450-snp164244	38.03–38.19	63.05	3.26	3.0	10.1	snp060453
LG28	qFCE3-28	snp050416-snp173574	38.56–39.20	63.05	3.12	3.0	9.7	snp247094
LG28	qFCE4-28	snp094907-snp190172	39.67–40.04	63.05	3.13	3.0	9.7	snp094899
LG28	qFCE5-28	snp105167-snp204381	42.27–42.67	63.05	3.05	3.0	9.7	snp137852
LG28	qFCE6-28	snp025842-snp168930	43.09–43.28	63.05	3.28	3.0	10.2	snp164078
LG28	qFCE7-28	snp031019-snp123627	43.46–43.86	63.05	3.05	3.0	9.5	snp173520
LG28	qFCE8-28	snp006951-snp008550	44.52–44.78	63.05	3.44	3.0	10.6	snp171092
LG32	qFCE1-32	snp076503-snp004530	15.07–16.29	70.72	3.40	3.0	11.0	snp004531
LG32	qFCE2-32	snp004530-snp066978	16.29–20.45	70.72	3.49	3.0	12.1	snp004486
LG32	qFCE3-32	snp066980-snp157023	21.35–23.59	70.72	3.04	3.0	9.5	snp157021
LG32	qFCE4-32	snp189518-snp085154	31.09–32.64	70.72	3.13	3.0	9.8	snp193531

*Represents the chromosome-wide significance LOD, threshold at *p* < 0.05.

### Candidate Gene Identification

To further identify potential causative genes, we screened the genome and collected protein-coding genes at the nearest SNP loci from each QTL. A total of nine genes associated with feed conversion efficiency in nine QTL were identified (Table 5). These candidate protein-coding genes were annotated by GO. We identified a gene, acetyl-CoA carboxylase alpha (*ACACA*), from the qFCE1-9 on LG9. We also identified two genes, SR-related CTD-associated factor 4 (*SCAF4*) and solute carrier family 2 member 5 (*SLC2A5*), from the qFCE2-20 and qFCE3-20 on LG20, respectively. On LG28, we identified three genes at three QTL. At the qFCE1-28, we identified a tenomodulin (*TNMD*) gene of which it has been reported that its polymorphisms were associated with adiposity and also with glucose metabolism in men (Tolppanen et al., 2007). We further identified a gene, protocadherin 1 (*PCDH1*), at the qFCE3-28. We also recognized a forkhead box O (*FOXO*) gene from the qFCE8-28. This gene encodes a protein that is regulated by factors involved in growth and differentiation indicating it plays a role in these processes (Wang et al., 2009; Zhu et al., 2010; Huo et al., 2014; Watamoto et al., 2019). We also identified three genes, argonaute RISC component 1 (AGO1), free fatty acid receptor 3-like (*FFAR3*), and AT rich interactive domain 1A (*ARID1A*) at qFCE1-32, QTL qFCE2-32, and qFCE3-32 on LG32, respectively.

**TABLE 5:** Summary of candidate genes for FCE trait in mirror carp.

QTL name	Chr	Associated SNPs	SNP location (bp)	Carp gene Id	Gene location (bp)		Gene name	Annotation
					From	To		
qFCE1-9	LG9	snp108444	22,188,096	CAFS_CommonC_G_091,796	22,185,301	22,207,580	*ACACA*	Acetyl-CoA carboxylase alpha
qFCE2-20	LG20	snp145912	13,067,352	CAFS_CommonC_G_057,517	13,064,285	13,078,405	*SCAF4*	SR-related CTD-associated factor 4
qFCE3-20	LG20	snp145909	14,807,639	CAFS_CommonC_G_057,228	14,795,909	14,807,894	*SLC2A5*	Solute carrier family 2 member 5
qFCE1-28	LG28	snp247112	10,256,532	CAFS_CommonC_G_063,176	10,254,148	10,297,988	*TNMD*	Tenomodulin
qFCE7-28	LG28	snp173520	10,000,245	CAFS_CommonC_G_062,723	9,998,381	10,015,209	*PCDH1*	Protocadherin 1
qFCE8-28	LG28	snp171092	2,535,708	CAFS_CommonC_G_062,559	2,533,722	2,542,477	*FOXO*	Forkhead box O
qFCE1-32	290	snp004531	562,238	CAFS_CommonC_G_004,026	560,784	589,106	*AGO1*	Argonaute RISC component 1
qFCE2-32	290	snp004486	372,623	CAFS_CommonC_G_004,020	370,723	376,820	*FFAR3*	Free fatty acid receptor 3-like
qFCE3-32	LG32	snp157021	20,092,699	CAFS_CommonC_G_068,410	20,081,844	20,104,892	*ARID1A*	AT rich interactive domain 1A

## Discusion

In past decades, fish-breeding methodologies have developed from traditional selection to modern biotechnologies, such as marker-assisted selection (MAS) and molecular breeding (Yue, 2014; Tong and Sun, 2015; Ashton et al., 2017). Implementation of MAS requires DNA markers that are tightly linked to traits of interest by means of QTL mapping or association analysis (Lande and Thompson, 1990; Rezende et al., 2012). Most economically important traits in fish, such as growth, disease resistance, and sex are controlled by multiple genes known as QTL. Most of these QTL have minor effects, but several pyramided may have major effects on traits (Tong and Sun, 2015). Theoretically, if genes and genetic markers associated with traits of interest are identified, the genetic variants could be used as tools in MAS analyses (Tong and Sun, 2015).

Considering common carp as a traditional cultivated species and commercial value, it has always been an important mission for breeders to cultivate new varieties with better characters. Up to now, QTL studies in common carp have covered a wide range of traits including growth, cold tolerance, meat quality, muscle fiber-related, sex determination, etc (Sun and Liang, 2004; Zhang et al., 2011; Laghari et al., 2013; Peng et al., 2016; Zheng et al., 2016). Compared to other economic traits, QTL analyses for FCE traits in aquaculture fish are rarely reported due to the difficulty to measure the phenotypes for these traits (Lu et al., 2017; Pang et al., 2017; Pang et al., 2018). So, it is expected that more genetics studies on FCE traits will be considered so that molecular breeding strategies for these traits would be developed. In this study, we constructed a high-density and high-resolution genetic linkage map for Songpu mirror carp and performed the first fine-scale QTL mapping for FCE. A group of closely linked markers and potential candidate genes were identified, which open new opportunities for MAS implementation, which can ultimately accelerate breeding mirror carp for high feed conversion efficiency.

### Ultra-High Density Genetic Map for Mirror Carp

It is well known that the separation type of mapping population directly affects the efficiency of linkage map construction (Wei et al., 2019). The commonly used mapping families for the construction of linkage genetic maps include F_2_ and backcross (BC) families, double haploid (DH), and recombinant inbred lines (RIL). However, it is a big challenge to construct DH or RIL families in most teleost fish, and the constructions of F_2_ and BC families usually take a relatively long time (Peng et al., 2016). The F_1_ progeny displays many different types of segregation. So, Grattapaglia and Sederoff proposed using the F_1_ family as the mapping panel with a double pseudo-testcross strategy which has been successfully applied to genetic linkage map construction in many aquaculture species (Grattapaglia and Sederoff, 1994; Wu et al., 2010; Song et al., 2012; Peng et al., 2016). In this study, the parents of the F_1_ population were derived from the cultured families of mirror carp at the Hulan station of the Heilongjiang Fisheries Research Institute of the Chinese Academy of Fishery Sciences, and the genetic distances among these fish were estimated using a panel of polymorphic microsatellite markers. A male and a female mature fish (F_0_) which showed a relatively high genetic distance were used to generate an experimental family (F_1_) by artificial crossing. Therefore, the mapping family used in this study with a double pseudo-testcross strategy was suitable for the construction of a genetic linkage map.

Obviously, a high-quality genetic map is an essential tool for QTL mapping with high efficiency and accuracy. However, a limited number of traditional markers make it difficult to cover the whole genome (Wen et al., 2020). The SNP marker is the most abundant and polymorphic marker in the genome which is very suitable for high-density linkage map construction. As an effective way, the second-generation sequencing technology makes it possible to obtain a sufficient number of SNP markers for linkage map construction in a short time. However, the call rate and genotyping accuracy of SNPs are critically important for the construction of a high-quality linkage map, as it is well known that missing data and genotyping errors would lead to incorrect map orders (Hackett and Broadfoot, 2003).

Compared with other SNP genotyping approaches, Affymetrix Axiom SNP genotyping platform has become an effective solution for large-scale SNP genotyping as it has higher call rates (>99%) and higher accuracy and has been successfully applied to the construction of the linkage map and QTL analysis of multiple species (Andreas et al., 2013; Sushma et al., 2016; Higgins et al., 2018; Gong et al., 2019; You et al., 2019). Recently, two high-density genetic linkage maps have been constructed based on Affymetrix Axiom SNP genotyping data for channel catfish (*Ictalurus punctatus*) and Yellow River carp (*C. carpio*), presenting the highest density linkage maps in aquaculture species (Li et al., 2015; Peng, et al., 2016). In this study, we also chose the same high-throughput SNP genotyping platform for SNP genotyping and constructed a high-density and high-accurate linkage genetic map for mirror carp. In order to get high-quality makers, we used more stringent data filtering (missing value < 1%) than the former studies of high-density linkage map construction. As a result, a total of 28,416 high-quality SNP markers were successfully mapped. The genetic map covered 99.07% of the genome with a density of 0.33 cM average distance, which demonstrates its power in the detection of potential QTL associated with FCE in mirror carp at a fine scale.

### QTL Analysis and Candidate Genes

Most economically important traits of fish such as growth, disease resistance, and flesh quality are controlled by multiple genes, environmental factors, and their interactions (Song et al., 2012; Shao et al., 2015; Peng et al., 2016; Zheng et al., 2016). Phenotypic variation of these traits is thought to be caused by quantitative genetic variation that results from the segregation of alleles at multiple quantitative trait loci (QTL) (Mackay et al., 2009; Zhang et al., 2021). The purpose of QTL mapping is to understand the number and effect of genes that determine traits and to assist in the selection of breeding to accelerate the genetic improvement of important traits (Doerge, 2002; Naish and Hard 2008).

A number of studies looking for genetic mechanisms affecting feed efficiency, which involved numerous biological processes and functional pathways in livestock and poultry, have been reported using different methods (Sherman et al., 2009; Do et al., 2014; Ferreira et al., 2016; Silva et al., 2019; Li et al., 2020; Li et al., 2021). Compared to the livestock and poultry, it has been a challenge to map loci associated with feed efficiency because the phenotypic value of each individual is generally difficult to obtain in aquaculture species (Lu et al., 2017; Pang et al., 2017). To date, only a few QTL analyses associated with feed conversion efficiency have been reported in aquaculture species. Preliminary QTL results about FCE in fish are from Liu’s study, which used AFLP markers to construct a catfish genetic map and found a QTL associated with FCE (Liu, 2001). Zimmerman et al. revealed three QTL for the number of pyloric caeca in three LGs of rainbow trout, and this is an important index associated with FCE (Zimmerman et al., 2005; Tong and Sun, 2015). Recently, Lu et al. performed the QTL mapping of feed conversion rate (FCR) in mirror carp based on two mapping panels consisting of 92 and 68 samples and using 507 and 307 markers, respectively. As a result, 18 QTL affecting FCR were detected in two datasets (Lu et al., 2017). Pang et al. constructed a high-resolution genetic linkage map in a full-sib F_1_ family of crucian carp (*Carassius auratus*) consisting of 113 progenies with 8,460 SNP markers. Eight FCE-related QTL and seven candidate genes involved in energy metabolism, digestion, biosynthesis, etc were detected (Pang et al., 2017). Although QTL about feed efficiency traits in common carp have been reported, not all of the genetic variants of the traits have been captured as the sample size of the mapping populations and the number of markers used were relatively small. QTL mapping has a large positioning range and there are too many genes in the location range. So the candidate genes were difficult to be determined. Historically, in the absence of high throughput genotyping data, it was difficult to perform QTL fine mappings and candidate gene identification. In this study, we were able to take advantage of the common carp 250K SNP genotyping array and construct an ultra-high density linkage map for mirror carp, providing new insights into the FCE trait and related genes. In the present investigation, a total of 17 QTL associated with FCE were mapped on four linkage groups, explaining 8.9–15.9% of the PVE, and nine candidate genes related to FCE involved in multiple biological processes were identified in common carp.

Feed conversion efficiency is a complex trait, which involves many physiological processes such as feeding, digestion, biosynthesis, metabolism, and so on, and is driven by a series of biological pathways. In our study, nine candidate genes associated with FCE were identified in common carp. Among these genes, *ACACA* has been reported as a part of a single multifunctional polypeptide in eukaryotes, and plays a critical role in the metabolism of fatty acid biosynthesis (Abu-Elheiga et al., 1995). *ACACA* is a biotin-containing enzyme that catalyzes the carboxylation of acetyl-CoA to malonyl-CoA, the rate-limiting step in fatty acid synthesis (Brusselmans et al., 2005). *ACACA* may induce dysregulation of lipid metabolism in human and mouse is known to result in metabolic diseases, such as obesity and diabetes (Wakil and Abu-Elheiga, 2009), it may also result in severe metabolic disorders in lactating cows (Loor et al., 2007). Therefore, we speculated that *ACACA* may be directly associated with fat metabolism and may subsequently affect FCE in common carp. The protein encod SCAF4 containing a domain, which found in hepatocyte growth factor-regulated tyrosine kinase substrate (Hrs), plays a critical role in the recycling and involved in endocytic trafficking (Li et al., 2002; Lea et al., 2019). So, we consider that its molecular function may be related to the growth of common carp, and may have a specific function related to FCE. *SLC2A5* has been reported as a facilitated glucose/fructose transmembrane transport which is the key solute carrier in the carbohydrate metabolic process of glucose and gluconeogenesis (Berghe, 1996). It takes part in carbohydrate digestion and absorption and leads to a significantly enhanced rate of triglyceride synthesis. *SLC2A5* is also responsible for fructose uptake by the small intestine in mammals (Wu et al., 2009; Nomura et al., 2016). High expression of *SLC2A5* in the small intestine in rats or mice leads to increased fructose absorption (Wright et al., 2007). A recent study showed that a higher expression level of *SLC2A5* was related to higher feed conversion efficiency-related traits (Diniz et al., 2020). Hence, we suggested that this gene was associated with food digestion, carbohydrate metabolic processing, and further influences on FCE in common carp. *TNMD* expression is highly affected by obesity, adipose tissue location, and weight loss in human adipose tissue, indicating that *TNMD* may play a role in adipose tissue function (Saiki et al., 2009). Therefore, we suggested that it may be associated with FCE by intervening in influence energy deposition and fat accumulation in common carp. The protein encoded by this gene has a domain associated with the catalytic domain of sugar utilizing enzymes, including maltooligosyl trehalose synthase, glycogen branching enzyme, glycogen debranching enzyme, isoamylase, etc, which endows PCDH1 with the capability to play several functions in different pathways (Aron et al., 2017). So, we suggested that it takes part in digestion and absorption and might be related to feed efficiency in common carp. FOXO is also involved in lipid metabolism and has been reported as one of the important candidate genes associated with obesity and body mass in human (Kim et al., 2006). The mRNA levels of FOXO isoforms in rat livers were altered in response to fasting and re-feeding, which suggests that the genes respond differently to nutritional and hormonal factors (Imae et al., 2003). Hence, we suggested that FOXO was associated with fat accumulation and growth in common carp. AGO1 encodes a member of the argonaute family of proteins which can affect cell proliferation, motility, and apoptosis in human (Parisi et al., 2011). Tang et al. (2020) report that AGO1 participates in a mechanism that controls adipose tissues, insulin sensitivity, and whole-body metabolic state. They found that when challenged with an obesity-inducing high-fat high-sucrose (HFHS) diet, AGO1-knockout mice displayed significantly lower body weight gain and lower body fat, improved insulin sensitivity, and enhanced energy expenditure. In human donors with obesity or type 2 diabetes mellitus, AGO1 is expressed at higher levels than in healthy controls, which also supports the role of this pathway (Tang et al., 2020). So, we indicate it is also possibly associated with digestive and metabolic functions in common carp. *FFAR3* is a G protein-coupled receptor that is activated by a major product of dietary fiber digestion, the short-chain fatty acids (*SCFAs*), which are an essential energy source and signaling molecules that regulate various cellular processes and physiological functions (Stoddart et al., 2008; Zaibi et al., 2010). Recent studies have shown that these receptors are involved in the lipid metabolism in various tissues and play an important role in the absorption of nutrients in animal intestine (Hara et al., 2013; Ichimura et al., 2014; Lu et al., 2015; Meza-Cuenca et al., 2018). Therefore, *FFAR3* may be a promising candidate gene for FCE in common carp, and further functional approaches are necessary to validate. *ARID1A* has been reported to affect cholesterol synthesis as well as glycogen metabolism-related proteins levels in human and could play a significant role to help cell proliferation and inhibit cell apoptosis (Flores-Alcantar et al., 2011; Bosse et al., 2013; Goldman et al., 2016). *ARID1A* deletion in isolated hepatocytes directly leads to free fatty acid-induced lipid accumulation and insulin resistance in mice. These findings reveal a new mechanism underlying the role of *ARID1A* in glucose and lipid metabolism (Qu et al., 2019). *ARID1A* has also been reported to have a positive correlation of expression with the body mass trait in human (Keildson et al., 2014; Giri et al., 2018). Thus, we speculate that *ARID1A* plays similar roles in regulating lipid accumulation and body mass in common carp.

Obviously, the QTL and genes detected in this study need to be further verified for their functional relatedness to FCE in the future. These novel findings would be used for future genetic and genomic researches of FCE traits, thereafter providing essential markers of MAS breeding for the potential improvement of feed efficiency in mirror carp.

## Conclusion

In conclusion, taking advantage of the common carp 250K SNP genotyping array, high throughput genotyping data were accurately and efficiently collected from a mirror carp mapping family and used to construct a high-density, high-resolution genetic linkage map for mirror carp. This map processes the highest marker density among all the constructed genetic maps in mirror carp. Based on this valuable genetic map, fine-scale QTL mapping of FCE was performed and candidate functional genes were also identified. These candidate genes were identified as functionally related to lipid metabolism, carbohydrate metabolism, energy deposition, fat accumulation, digestion, regulating growth, and cell proliferation and differentiation. The present high-density genetic map and mapping results provide a basis for further genetic research of feed conversion efficiency and facilitate future MAS breeding for the feed conversion efficiency trait in common carp.1

## Data Availability

The datasets presented in this study can be found in online repositories. The names of the repository/repositories and accession number(s) can be found below: https://www.ncbi.nlm.nih.gov/, GCF_018340385.
