# Supplementary material for: A High-Density Genetic Linkage Map and Fine Mapping of QTL For Feed Conversion Efficiency in Common Carp (Cyprinus carpio)
Source: Front Genet. 2021 Nov 12;12:778487. doi: 10.3389/fgene.2021.778487 (PMC8633483; doi:10.3389/fgene.2021.778487)
Supplement: Supplementary file 2 [file Table1.DOCX]

Table S1: The composition of carp feed.

| Crude protein (%) ≥ | Crude fiber  (%) ≤ | Crude fat  (%) ≥ | Crude ash  (%) ≤ | Calcium  (%) | Total phosphorus (%) ≥ | Sodium chloride (%) | Water  (%) ≤ | Lysine  (%) ≥ |
| --- | --- | --- | --- | --- | --- | --- | --- | --- |
| 32.0 | 12.0 | 8.0 | 15.0 | 0.50–2.00 | 1.00 | 0.30–2.00 | 14.0 | 1.40 |
